# Rationale, study design, and analysis plan of the Alveolar Recruitment for ARDS Trial (ART): Study protocol for a randomized controlled trial

**DOI:** 10.1186/1745-6215-13-153

**Published:** 2012-08-28

**Authors:** The ART Investigators

**Affiliations:** 1 Research Institute – Hospital do Coração (IEP– HCor), Rua Abílio Soares 250, 12th floor, CEP: 04005-000, São Paulo, SP, Brazil

**Keywords:** Acute respiratory distress syndrome, Alveolar recruitment, PEEP, Mechanical ventilation, Clinical trials, Randomized

## Abstract

**Background:**

Acute respiratory distress syndrome (ARDS) is associated with high in-hospital mortality. Alveolar recruitment followed by ventilation at optimal titrated PEEP may reduce ventilator-induced lung injury and improve oxygenation in patients with ARDS, but the effects on mortality and other clinical outcomes remain unknown. This article reports the rationale, study design, and analysis plan of the Alveolar Recruitment for ARDS Trial (ART).

**Methods/Design:**

ART is a pragmatic, multicenter, randomized (concealed), controlled trial, which aims to determine if maximum stepwise alveolar recruitment associated with PEEP titration is able to increase 28-day survival in patients with ARDS compared to conventional treatment (ARDSNet strategy). We will enroll adult patients with ARDS of less than 72 h duration. The intervention group will receive an alveolar recruitment maneuver, with stepwise increases of PEEP achieving 45 cmH_2_O and peak pressure of 60 cmH_2_O, followed by ventilation with optimal PEEP titrated according to the static compliance of the respiratory system. In the control group, mechanical ventilation will follow a conventional protocol (ARDSNet). In both groups, we will use controlled volume mode with low tidal volumes (4 to 6 mL/kg of predicted body weight) and targeting plateau pressure ≤30 cmH_2_O. The primary outcome is 28-day survival, and the secondary outcomes are: length of ICU stay; length of hospital stay; pneumothorax requiring chest tube during first 7 days; barotrauma during first 7 days; mechanical ventilation-free days from days 1 to 28; ICU, in-hospital, and 6-month survival. ART is an event-guided trial planned to last until 520 events (deaths within 28 days) are observed. These events allow detection of a hazard ratio of 0.75, with 90% power and two-tailed type I error of 5%. All analysis will follow the intention-to-treat principle.

**Discussion:**

If the ART strategy with maximum recruitment and PEEP titration improves 28-day survival, this will represent a notable advance to the care of ARDS patients. Conversely, if the ART strategy is similar or inferior to the current evidence-based strategy (ARDSNet), this should also change current practice as many institutions routinely employ recruitment maneuvers and set PEEP levels according to some titration method.

**Trial registration:**

ClinicalTrials.gov Identifier: NCT01374022

## Background

Acute respiratory distress syndrome (ARDS) is a common problem in critically-ill patients, associated with in-hospital mortality between 41% and 58% [1-3] and reduced quality of life among survivors [4,5]. Although mechanical ventilation provides essential life support, it can worsen lung injury [6]. Mechanisms of ventilator-induced lung injury include regional alveolar overdistention, repetitive alveolar collapse with shearing (atelectrauma), and oxygen toxicity [7,8].

Ventilation with low tidal volumes (≤6 mL/kg) and targeting plateau pressures of 30 cmH_2_O or less improves survival compared to the use of high volumes (12 mL/kg), confirming the relevance of avoiding overdistention in ARDS [9]. Although this strategy improved care of ARDS patients, mortality is still unacceptably high [3]. Experimental data suggest that atelectrauma is prominent in ARDS [10,11] and may be a contributor to ARDS mortality [12]. Opening of collapsed lung tissue by recruitment maneuvers and preventing further collapse by using titration of PEEP may prevent atelectrauma. Maximum alveolar recruitment followed by PEEP titration is a relatively simple and widely available intervention.

Some studies demonstrated that maximum recruitment strategy, achieving PEEP of 45 cmH_2_O and peak pressure of 60 cmH_2_O, can fully recruit the lung and reverse hypoxemia in most ARDS patients, without major adverse events [13,14]. However, a systematic review of alveolar recruitment maneuvers found only four randomized trials and was inconclusive regarding the effect of recruitment maneuvers on survival and other patients’ relevant outcomes [15]. Recently a pilot study randomized 20 patients to an alveolar recruitment maneuver with progressive PEEP elevation up to 40 cmH_2_O and peak pressure of 55 cmH_2_O plus PEEP titrated according to peripheral oxygen saturation or to ARDSNet strategy [16]. There were a decrease in some systemic cytokines, and improvement in oxygenation and compliance. As expected, the trial was not powered to and did not show any difference in mortality and other clinical outcomes. Therefore, a trial with high methodological quality and power to assess whether maximum alveolar recruitment followed by ventilation with titrated PEEP improves clinical outcomes in ARDS patients is highly needed.

## Methods

### Objectives

Our primary objective is to determine if maximum alveolar recruitment associated with PEEP titrated according to the static compliance of the respiratory system (ART strategy) increases 28-day survival rate of patients with acute respiratory distress syndrome compared to conventional treatment (ARDSNet strategy).

Secondary objectives are to evaluate the effect of the ART strategy compared to ARDSNet strategy on the following outcomes: length of hospitalization; pneumothorax requiring chest tube at 7 days; barotrauma (any pneumothorax, pneumomediastinum, subcutaneous emphysema or pneumatocele >2 cm after randomization) at 7 days; ventilator-free days from days 1 to 28; intensive care unit, in-hospital, and 6-month survival.

### Study design

ART is a randomized, stratified, multicenter trial with allocation concealment and intention-to-treat analysis. Patients with ARDS will be treated with a stepwise maximum alveolar recruitment maneuver followed by ventilation with optimal PEEP (ART Strategy) *vs.* a conventional approach (ARDSNet Strategy). This is an event-guided trial which will end when 520 events (deaths within 28 days) are observed. Patients will be followed up to 6 months, although the main outcome is determined at the 28-day follow-up.

### Screening

Eligibility will be evaluated in two phases: screening phase and defining eligibility phase.

In the screening phase, patients will be considered for inclusion in the study if they are receiving invasive mechanical ventilation and have ARDS of less than 72 h duration. All of the following criteria should be met [17]: acute onset respiratory failure; bilateral pulmonary infiltrate on chest X-ray compatible with pulmonary edema; severe hypoxemia, defined as PaO_2_/FiO_2_ ≤200 in arterial blood gases for less than 72 h; absence of left atrial hypertension based on the medical team’s evaluation (clinical or echocardiographic signs); and presence of a risk factor for lung injury.

The following are exclusion criteria (exclusion if any one present): age <18 years; use of vasoconstrictor drugs in increasing doses over the past 2 h (norepinephrine increase ≥0,5 mcg/kg/min or dopamine increase ≥5 mcg/kg/min) or mean arterial pressure <65 mmHg; this may be a transient criterion, since patients meeting this criterion might be included later if hemodynamics improves; contraindications to hypercapnia such as intracranial hypertension or acute coronary syndrome; and undrained pneumothorax or subcutaneous emphysema.

While waiting for the consent of a legal representative or for at least 3 h, we suggest to ventilate patients using a conventional approach as follows [9]: volume-controlled mode, tidal volume of 4 to 6 mL/kg of predicted body weight to ensure plateau pressure ≤30 cmH_2_O, PEEP, and FiO_2_ adjusted according to the ARDSNet table (Table 1) to maintain SpO_2_ ≥88% and PaO_2_ ≥55 mmHg, flow of 60 L/min, descending waveform, inspiratory pause of 0.5 s, inspiratory to expiratory ratio (I:E) of 1:1 to 1:2, respiratory rate to keep PaCO_2_ between 35 and 60 mmHg. Alveolar recruitment maneuvers should be avoided. 

**Table 1:** **ARDSNet table of FiO**_**2**_**and PEEP values to keep SpO**_**2**_ **≥ 88% or PaO**_**2**_ **≥ 55 mmHg**

**FiO**_**2**_	**30%**	**40%**	**40%**	**50%**	**50%**	**60%**	**70%**	**70%**	**70%**	**80%**	**90%**	**90%**	**90%**	**100%**
PEEP	5	5	8	8	10	10	10	12	14	14	14	16	18	18-24

Predicted body weight should be calculated for all patients according to the formula:

(1) $$\text{Men}:\text{Predicted}\;\text{body}\;\text{weight}\left(\text{kg}\right)=50+2.3\left(\left(\text{height}\left[\text{cm}\right]*0.394\right)-60\right)$$

(2) $$\text{Women}:\text{Predicted}\;\text{body}\;\text{weight}\left(\text{kg}\right)=45.5+2.3\left(\left(\text{height}\left[\text{cm}\right]*0.394\right)-60\right)$$

### Defining eligibility

Right after obtaining informed consent, ventilator will be set as described above (if the recommended ventilation were not set yet) and FiO_2_ will be adjusted to 100% and PEEP to 10 cmH_2_O (except if previous PEEP were ≥16 cmH_2_O; in this case PEEP will be maintained). Arterial blood gases will be measured after 30 min.

Patients will be considered eligible if the PaO_2_ measured with FiO_2_ = 100% and PEEP = 10 cmH_2_O (or ≥16 cmH_2_O) is 200 mmHg or less, and less than 72 h have been spent since the first time a PaO_2_/FiO_2_ ≤200 was determined.

### Criteria for withdrawal of patients from the trial

The withdrawal of a patient from the study will occur only if consent is withdrawn by the patient, his/her legal representative, or the patient’s primary care physician.

Treatment should be discontinued if the patient is sufficiently unstable to contraindicate the continued use of high PEEP levels. The necessary measures to minimize instability and adverse effects caused by the use of high PEEP levels should be implemented as deemed appropriate by the medical team. However, patient follow-up will proceed normally, that is, the patient will not be excluded from follow-up and analyses.

### Randomization and allocation concealment

Patients will be randomized in a 1:1 ratio to the ART strategy or the ARDSNet strategy.

The random allocation list was generated in blocks (number of treatments per block will be kept confidential to avoid prediction of future patients’ allocation) and was stratified by investigator center age, and PaO_2_/FiO_2_ ratio (≤100 or >100). Allocation concealment will be maintained by means of a web-based central, automated randomization system, available 24 h a day (ACT-Clinic), developed by a team of programmers and investigators from the Research Institute at Hospital do Coração (IEP-HCor). The group to which the patient will be allocated will only be disclosed after patient enrollment information is recorded in the electronic system. This prevents the investigator and the medical team from predicting to which treatment group the patient will be allocated. To include a patient in the study, investigators must simply access the IEP-HCor website ( https://servicos.hcor.com.br/iep/estudoclinico), log in with individual username and password, and fill in a short medical record form.

### Interventions

Table 2 summarizes the procedures that will be used for ART and ARDSNet groups in this study.

**Table 2:** **Summary of mechanical ventilation procedures in the ART strategy group*vs.*ARDSNet strategy group**

**Procedure**	**ART strategy: maximum alveolar recruitment maneuver associated with PEEP titration**	**ARDSNet strategy**
Alveolar recruitment maneuver	Yes (see Figure 1)	No
Ventilation mode	Volume-controlled	Volume-controlled
Target plateau pressure and driving pressure	Plateau ≤30 cmH_2_O	Plateau ≤30 cmH_2_O
Target tidal volume	4 to 6 mL/kg of predicted body weight	4 to 6 mL/kg of predicted body weight
Respiratory rate and pH goal	6 to 35/min, adjusted for pH ≥ 7.30 if possible	6 to 35/min, adjusted for pH ≥ 7.30 if possible
I:E ratio	1:1 to 1:2; flow 60 L/min; inspiratory pause 0.5 s	1:1 to 1:2; flow 60 L/min; inspiratory pause 0.5 s
Oxygenation goals		
PaO_2_	60 to 80 mmHg	55 to 80 mmHg
SpO_2_	90 to 95%	88 to 95%
PEEP and FiO_2_ adjustment	PEEP titration 2 cmH_2_O above PEEP value associated with maximum compliance. FiO_2_ titration adjusted according to oxygenation goals	According to PEEP/FiO_2_ combination table
Weaning	After 24 h with PaO_2_/FiO_2_ ≥ 300 (or stable/ascending) start weaning from PEEP 2 cmH_2_O every 8 h. Consider pressure support ventilation after PEEP ≤ 14 cmH_2_O. Spontaneous ventilation test in PS = 5 cmH_2_O and PEEP = 5 cmH_2_O. Routine use of NIV immediately after extubation is encouraged	Weaning from PEEP according to table of PEEP and FiO_2_ combinations. Consider pressure support ventilation after PEEP ≤ 14 cmH_2_O. Spontaneous ventilation test in PS = 5 cmH_2_O and PEEP = 5 cmH_2_O. Routine use of NIV immediately after extubation is encouraged

### ART strategy (maximum alveolar recruitment maneuver plus PEEP titration)

**Figure 1:**
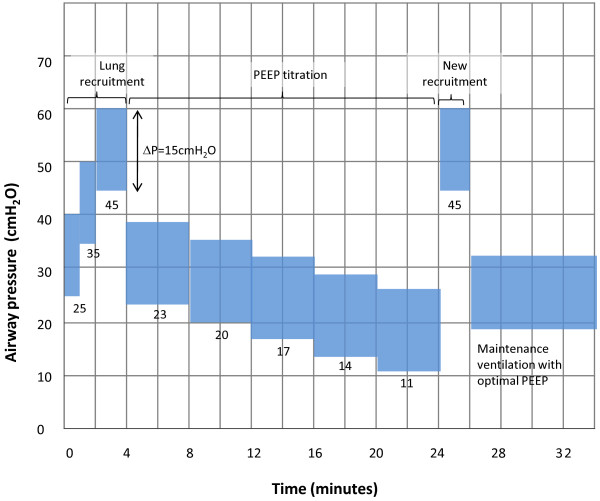
ART strategy: maximum alveolar recruitment maneuver associated with PEEP titration.

If patients are assigned to the ART strategy, the following steps should be observed (Figure 1):

1. Preparation for the recruitment maneuver

Patients will be sedated, paralyzed (with a neuromuscular blocker), and kept in supine position. Closed endotracheal suction system will be installed. Monitoring will be provided with at least: heart rate, cardiac rhythm, periferic oxygen saturation, and blood pressure (preferably invasive method). Hypovolemia will be corrected by crystalloid or colloid infusion until variation of arterial pulse pressure ≤13%, or central venous pressure of >10 cmH_2_O if variation of arterial pulse pressure measurement is not available. If the variation of arterial pulse pressure method is used, we recommend to transiently setting tidal volume to 8 mL/kg for 15 min before measurements

2. Maximum alveolar recruitment maneuver Mechanical ventilator will be set to pressure-controlled mode with FiO_2_ of 100%; respiratory rate of 10/min and I:E ratio of 1:1. Alveolar recruitment maneuver steps are described below:

Recruitment starts with PEEP of 25 cmH_2_O and driving pressure of 15 cmH_2_O. These parameters will be maintained for 1 min;

Following this, PEEP will be increased to 35 cmH_2_O with other parameters maintained for 1 min;

Lastly, PEEP will be increased to 45 cmH_2_O with other parameters maintained for 2 min.

 Recruitment will be terminated if one or more of the following signs of clinical deterioration are observed: heart rate >150 or <60 bpm; decrease of mean arterial blood pressure <65 mmHg or decrease of systolic blood pressure <90 mmHg; decrease of SpO_2_ <88% for >30 s; acute atrial fibrilation, atrial flutter or ventricular tachycardia.

If recruitment is interrupted, physicians should proceed to PEEP titration, but should not repeat recruitment after titration (see next section ‘PEEP titration and new recruitment’). If a patient remains unstable while PEEP is titrated, then PEEP titration should be interrupted and the patient should be placed on the ARDSNet protocol. In this case, recruitment should be considered later if the patient’s condition stabilizes.

3. PEEP titration and new recruitment

Right after completing recruitment, PEEP will be set to 23 cmH_2_O. Ventilatory mode will be set to volume-controlled, tidal volume to 5 mL/kg of predicted body weight, respiratory rate to 20/min, flow to 30 L/min (square wave) and FiO_2_ to 100%. After 4 min, static compliance of the respiratory system will be calculated and recorded (inspiratory pause ≥2 s required to reach plateau pressure). In sequence, PEEP will be decreased in steps of 20, 17, 14, and 11 cmH_2_O and the corresponding static compliance of the respiratory system will be recorded after each 4 min.

Optimal PEEP will be the PEEP associated with the best static compliance plus 2 cmH_2_O. If a ‘plateau’ of best compliance is achieved, that is, more than one PEEP level associated with the best compliance, then the higher of the PEEP levels within the ‘plateau’ plus 2 cmH_2_O is considered the optimal PEEP.

If falls in compliance are verified in two consecutive steps, the PEEP level with the highest compliance plus 2 cmH_2_O is the optimal PEEP. In this case, there is no need to measure compliance on lower PEEP levels.

After the PEEP titration phase, a new alveolar recruitment will be performed. The mechanical ventilator will be reset to pressure-controlled mode; respiratory rate to 10/min; I:E ratio to 1:1, driving pressure to 15 cmH_2_O, FiO_2_ to 100% and PEEP adjusted to 45 cmH_2_O. These parameters will be maintained for 2 min.

4. Maintenance ventilation

Following the new alveolar recruitment, the maintenance ventilation will be set: volume-controlled mode, tidal volume of 5 mL/kg of predicted body weight. If plateau pressure >30 cmH2O, reduce to 4 mL/kg of predicted body weight. Minimum and maximum tidal volumes are 4 mL/kg and 6 mL/kg of predicted body weight. Flow of 60 L/min, descending waveform, inspiratory pause of 0.5 s, I:E ratio of 1:1 to 1:2, respiratory rate to maintain the same minute ventilation prior to randomization, PEEP adjusted to optimal PEEP (PEEP value at maximum compliance plus 2 cmH_2_O) and FiO_2_ adjusted for SpO_2_ ≥90% and ≤95%.

5. When to repeat a recruitment maneuver

Repetition of the maximum alveolar recruitment maneuver will be considered only when the initial maneuver is successful, which we defined as an increase of PaO_2_/FiO_2_ ratio >100 after maneuver.

If the initial maneuver is successful, it will be repeated in two situations:

Every 24 h if PaO_2_/FiO_2_ ratio is <250 and a decrease in PaO_2_/FiO_2_ ratio >50 occurs. After the recruitment maneuver, PEEP should be set at the value it was before plus 2 cmH_2_O. That is, there is no need to titrate PEEP again.

If an accidental disconnection of the respiratory circuits occurs and PEEP is ≥12 cmH_2_O. After the recruitment maneuver, PEEP should be set at the same level it was before disconnection. There is no need to titrate PEEP again.

6. Adjustments in tidal volume and respiratory rate

Adjustments in tidal volume and respiratory rate are the same for ART group and ARDSNet group, and is described below.

Respiratory rate and tidal volume must be adjusted to achieve the arterial pH goal: between 7.30 and 7.45. The pH is measured when clinically indicated.

#### Management of alkalemia and acidemia

Alkalemia (pH >7.45): reduce respiratory rate, if possible

Mild acidemia (7.15 ≤ pH <7.30):

– If PaCO_2_ ≤ 40 mmHg, consider sodium bicarbonate and, if possible, treat the cause of metabolic acidosis

– If PaCO_2_ > 40 mmHg:

○ Increase respiratory rate up to a maximum of 35 aiming a pH >7.30 or PaCO_2_ <40 mmHg, whichever occurs first. If there is associated metabolic acidosis, it should also be managed.

○ If the respiratory rate =35 and pH is between 7.15 and 7.30, there is no need of additional measures.

Severe acidemia (pH <7.15):

If PaCO_2_ ≤ 40 mmHg, consider sodium bicarbonate and, if possible, treat the cause of metabolic acidosis.

If PaCO_2_ > 40 mmHg

PEEP weaning can start 24 h after the initial alveolar recruitment maneuver. PEEP can be decreased 2 cmH_2_O each 8 h as long as the PaO_2_/FiO_2_ ratio are >300. In case of not achieving PaO_2_/FiO_2_ ratio >300 after alveolar recruitment, PEEP can be decreased 2 cmH_2_O each 8 h if PaO_2_/FiO_2_ ratio is similar or greater than the day before.

Other than the PEEP weaning procedure described above, the rest of the weaning method is equal in the ART and ARDSNet groups, and is described below.

Pressure support (PS) ventilation can be initiated in alert patients when PEEP ≤14 cmH_2_O. Start with PS of 10 cmH_2_O or less to achieve tidal volume of 6 mL/kg of predicted body weight. PS ventilation can be reduced from 2 to 4 cmH_2_O twice daily as long as respiratory frequency is <28 breaths per min (and there are no other signs of discomfort). In patients with signs of discomfort (for example, those with ≥30 breaths per minute) investigators should consider other causes (such as pain or anxiety) before increasing PS. If PS over 14 cmH_2_O is needed, then volume-controlled ventilation will be resumed.

Daily assessments to attempt the spontaneous breathing test will be performed preferably during the morning. The criteria shown on Table 3 will be considered to start the spontaneous breathing test. Spontaneous ventilation test will be performed using PS mode, with PEEP of 5 cmH_2_O, PS of 5 cmH_2_O, for 30 min. Criteria to diagnose failure in spontaneous ventilation test are presented in Table 4.

**Table 3:** Type of assessment and criteria for performing a spontaneous breathing test

**Clinical assessment**	**Improvement of acute process (ARDS and associated conditions) leading to intubation and mechanical ventilation**
	Patient is alert and cooperative
	Chest pain is controlled
	Adequate cough (moderate to high strength)
	Absence of excessive tracheobronchial secretion
	No signs of respiratory distress:
	Nostril flaring
	Use of accessory muscles of respiration (suprasternal and/or intercostal retraction)
	Paradoxical movements of the chest/abdomen
Objective measurements	Respiratory stability: oxygenation
	PEEP ≤10 cmH_2_O
	Support pressure ≤10 cmH_2_O
	PaO_2_/FiO_2_ ≥250 (consider weaning if ≥150)
	SpO_2_ > 90% under FiO_2_ ≤40%
	Respiratory stability: function
	Respiratory rate ≤35 breaths/min
	Minute volume < 10 L/min
	Respiratory rate/tidal volume (L) < 105 breath/min/L
	No significant respiratory acidosis (pH ≥7.25)
	Cardiovascular stability
	Heart rate <140 bpm
	Systolic blood pressure > 90 and <160 mmHg
	Without vasoconstrictor/inotropic drugs (or low doses)
	Neurological stability
	Patient alert and cooperative - SAS 4 (acceptable: slightly drowsy patient (SAS 3) slightly agitated (SAS 5))

**Table 4:** Type of assessment and criteria for failure of the spontaneous breathing test

**Clinical assessment**	**Agitation, excessive anxiety, or depressed level of consciousness**
	Major sweating
	Cyanosis
	Signs of respiratory distress:
	Nostril flaring
	Use of accessory muscles of respiration (suprasternal and/or intercostal retraction)
	Paradoxical movements of the chest/abdomen
Objective measurements	Respiratory instability: oxygenation
	SpO_2_ < 90%
	Respiratory instability: function
	Respiratory rate > 35 breaths/min or increase >10 breaths/min
	Respiratory rate/tidal volume (L) <105 breath/min/L
	If arterial blood gases measured:
	pH <7.25
	PaCO_2_ > 50 mmHg or increase >8 mmHg
	Cardiovascular instability
	Heart rate <140 bpm
	Systolic blood pressure < 90 and >160 mmHg
	Onset of arrhythmias (for example, frequent ventricular extrasystole)

Patients who pass the spontaneous breathing test can be extubated. Cuff leak test is optional. Systemic steroids for patients intubated for long periods, with the aim to prevent upper airway obstruction after extubation, are also optional. Non-invasive ventilation should be considered for all patients. This is strongly recommended for patients at high risk of extubation failure, such as: patients who do not meet all the criteria for extubation (for example, respiratory rate/tidal volume (L) ≥105 breaths/min/L); and patients who failed the spontaneous breathing test at least once.

Increase respiratory rate to 35. If there is associated metabolic acidosis, it should also be managed.

If respiratory rate =35, pH <7.15, and PaCO_2_ >40 mmHg, increase tidal volume in steps of 1 mL/kg, up to 6 mL/kg of predicted body weight. In this condition, the plateau pressure goal of 30 cmH_2_O can be exceeded.

If respiratory rate =35, pH <7.15, PaCO_2_ >40 mmHg, and tidal volume is 6 mL/kg of predicted body weight, increase tidal volume to 7 mL/kg of predicted body weight.

If the situation remains unresolved (respiratory rate =35, pH <7.15, PaCO_2_ >40 mmHg, and tidal volume is 7 mL/kg of predicted body weight) increase tidal volume to 8 mL/kg of predicted body weight.

7. Refractory hypoxemia

Refractory hypoxemia is defined as a PaO_2_ < 55 mmHg or SpO_2_ < 88% with FiO_2_ = 100%. The following sequential actions should be taken for patients presenting with refractory hypoxemia:

Prone position;

If the patient does not improve, then start inhaled nitric oxide, if available, beginning with 5 ppm and increasing in steps of 5 ppm until there is improvement in oxygenation;

Final step is to initiate extracorporeal membrane oxygenation (ECMO) if available.

8. Weaning from mechanical ventilation

### ARDSNet strategy (conventional ventilation)

1. Maintenance ventilation

If patients are assigned to the ARDSNet strategy, no alveolar recruitment will be performed. The conventional mechanical ventilation strategy that will be used in this group has been described previously [9,18]. Initial ventilator settings will be: 

Volume-controlled mode;

Plateau pressure ≤30 cmH_2_O;

Tidal volume of 5 mL/kg of predicted body weight. If plateau pressure >30 cmH_2_O, reduce to 4 mL/kg of predicted body weight. Minimum and maximum tidal volumes are 4 mL/kg and 6 mL/kg of predicted body weight;

Respiratory rate will be adjusted with the aim of maintaining the same minute volume recorded before study entry. Maximum respiratory rate will be 35/min;

Flow 60 L/min;

Descending inspiratory flow;

Inspiratory pause 0.5 s;

I:E ratio between 1:1 a 1:2;

PEEP and FiO_2_ adjusted according to the ARDSNet (Table 1) aiming to keep the oxygenation goals: SpO_2_ between 88% and 95%, and PaO_2_ between 55 mmHg and 80 mmHg.

2. Adjustments in tidal volume and respiratory rate

These are the same as described for the ART Group above.

3. Refractory hypoxemia

The definition of refractory hypoxemia is the same as for the ART group, that is, a PaO_2_ < 55 mmHg or SpO_2_ < 88% with FiO_2_ = 100%.

The sequence of actions to be taken for patients presenting with refractory hypoxemia is similar, except for the first two steps, as is describe below:

Increase PEEP up to 24 cmH_2_O and FiO_2_ to 100% (as in the right end of the ARDSNet table (Table 1));

If no improvement is achieved, than PEEP should be increased 2 to 5 cmH_2_O each step up to 34 cmH_2_O or until PaO_2_ is between 55 mmHg and 80 mmHg or SpO_2_ is 88%; to 95%.

If no improvement is achieved, PEEP should be lowered to 24 cmH_2_O, and the sequence described above to manage refractory hypoxemia in the ART group should be followed. First step, is to put the patient in the prone position;

If the patient does not improve, then start inhaled nitric oxide, if available, beginning with 5 ppm and increasing in steps of 5 ppm until there is improvement in oxygenation;

Final step is to initiate ECMO if available.

4. Weaning from mechanical ventilation

Weaning of PEEP and FiO_2_ is done following the ARDSNet table (Table 1).

Other aspects of weaning are the same as described above for the ART group.

### Blinding

Since the intervention will be administered to critically-ill patients on mechanical ventilation (that is, mostly sedated), blinding of these patients is not necessary. Because this is a non-pharmacological intervention, blinding of the medical team is not feasible. There is no need for a committee to validate the primary study outcome (28-day survival), and therefore outcome adjudicators will not be blinded.

### Outcomes

The primary outcome of the ART is 28-day survival. The secondary outcomes are: (1) length of ICU stay; (2) length of hospital stay; (3) pneumothorax requiring chest tube at 7 days; (4) barotrauma (any pneumothorax, pneumomediastinum, subcutaneous emphysema, or pneumatocele > 2 cm after randomization) at 7 days; (5) mechanical ventilation-free from days 1 to 28; (6) ICU survival; (7) in-hospital survival; and (8) 6-month survival.

### Data collection and management

Study follow-up and the variables that will be collected are described below.

#### *Screening and eligibility data (Day 0)*

Patient’s initials, gender, date of birth

Verification of ARDS criteria

Screening inclusion and exclusion criteria

Respiratory variables (tidal volume, plateau pressure, total respiratory rate, PEEP, FiO_2_) while awaiting informed consent

Final eligibility criteria:

PaO_2_ at FiO_2_ of 100% and PEEP of 10cmH_2_O (or greater if previous PEEP were ≥16cmH_2_O)

Estimated time from onset of ARDS (onset of ARDS based on arterial blood gases until randomization)

#### *Baseline Data (Day 0)*

The following data will be recorded at the baseline visit:

Weight (measures with a weighing scale)

Height

SAPS 3 (at ICU admission)

Respiratory variables (Tidal volume, Plateau pressure, Total respiratory rate, PEEP, FiO_2_)

Sequential organ failure assessment (SOFA)

Cause of ARDS

Days of intubation prior randomization

#### *Treatment Data (1 h after start of intervention)*

The following data regarding treatment will be assessed for patients randomized to ART strategy:

Alveolar recruitment (for the group treated with maximum alveolar recruitment)

Maximum PEEP reached

If maximum alveolar recruitment is interrupted, the reason provided (list of criteria for interruption)

The following data regarding treatment will be assessed for all patients:

Respiratory variables of maintenance ventilation (Tidal volume, Plateau pressure, Total respiratory rate, PEEP, FiO_2_, PaO_2_, PaCO_2_, Arterial pH)

Hemodynamic variables (Heart rate, Mean blood pressure, Use of noradrenaline and dopamine)

#### *1-Day Follow-Up*

Respiratory variables of maintenance ventilation (Tidal volume, Plateau pressure, Total respiratory rate, PEEP, FiO_2_, PaO_2_, PaCO_2_, Arterial pH)

Water balance and weight (weighing scale)

#### *3-Day Follow-Up*

Respiratory variables of maintenance ventilation (Tidal volume, Plateau pressure, Total respiratory rate, PEEP, FiO_2_, PaO_2_, PaCO_2_, Arterial pH)

Water balance and weight (weighing scale)

#### *7-day follow-up*

Vital status at day 7. If patient dies before day 7, the following variables should be collected:

 Date of death

 Death due to refractory hypoxemia

 Death due to refractory respiratory acidosis

 Death due to refractory barotrauma

 Number of days on mechanical ventilation

Respiratory variables of maintenance ventilation (Tidal volume, plateau pressure, total respiratory rate, PEEP, FiO_2_, PaO_2_, PaCO_2_, arterial pH)

Water balance and weight (weighing scale)

Co-interventions during the period (use of/number of days using neuromuscular blockers; use of/number of days using continuous infusion of sedatives; use of/number of days using continuous infusion of narcotics; use of/number of days using noradrenaline or dopamine; use of/number of days using corticoids; rescue therapies for refractory hypoxemia: prone position, nitric oxide, high frequency oscillatory ventilation, ECMO)

Pneumothorax requiring chest tube drainage during the period

Barotrauma (any pneumothorax, pneumomediastinum, subcutaneous emphysema, or pneumatocele > 2 cm after randomization) during the period

#### *Hospital discharge*

Date of ICU discharge and vital status at ICU discharge

Date of hospital discharge and vital status at hospital discharge

#### *28-day follow-up*

Days on mechanical ventilation (considering the first 28 days after randomization)

Vital status and date of death (for patients who died)

#### *6-month follow-up*

Vital status and date of death (for patients who died)

### Clinical Data Management System (CDMS) and quality control

Besides the 24-h concealed randomization, the ACT Clinic will provide data entry, data cleaning, and exportation for analysis. The system will also provide reports on the status of the study forms (completed forms, overdue forms), weekly study recruitment by center, and graphs of observed and expected cumulative recruitment.

Several procedures will assure data quality, including: (1) all investigators will attend a training session before the start of the study to standardize procedures, including data collection; (2) the investigators may contact the Study Coordinating Center to solve issues or problems that may arise; (3) data entry into the ACT-Clinic is subject to various checks for missing data, plausible, possible or non-permitted value ranges, and logic checks. Problems are informed by the system at the time of data entry; (4) statistical techniques to identify inconsistencies will be applied periodically (about every two weeks). The centers will be notified of the inconsistencies and asked to correct them; (5) statistical routines to identify fraud will be conducted periodically (every 90 days); (6) on-site monitoring will be conducted during study conduction; (7) the coordinating center will review detailed reports on screening, enrolment, follow-up, inconsistencies, and completeness of data. Immediate actions will follow to solve problems that arise.

### Sample size

ART is an event driven study designed to last until 520 events (deaths within 28 days) are observed. This number of events is sufficient to detect a hazard ratio of 0.75 (that is, relative reduction in event rate of 25%), considering a type I error of 5%, 90% power, and a similar allocation of subjects to each group. Considering an event rate in the control group of 36% (mean proportion of deaths in randomized studies conducted after 1994, when the definition of ARDS was first standardized) [3] we expect that about 1,620 patients will be needed to achieve the number of events planned. However, since this is an event driven study, the total sample size can vary depending on the event rate in the experimental and control groups.

### Statistical analysis plan

All analysis will follow the intention-to-treat principle. Survival within 28 days (primary outcome) in both groups will be assessed using Kaplan-Meier curves and Cox proportional hazard models, without adjustment for other co-variates. Treatment effects on length of ICU stay, hospital stay, and number of mechanical ventilation-free days at 28 days will be analyzed using Mann–Whitney tests. Occurrence of pneumothorax and barotrauma will be evaluated using chi-square tests; ICU and in-hospital mortality will be evaluated with chi-square tests; 6-month survival will be analyzed using Kaplan-Meier curves and Cox proportional hazards models. Treatment effect on 28-day survival will be analyzed in the following subgroups: (1) PaO_2_/FiO_2_ ≤100 *vs.* >100; (2) SAPS 3 score <50 *vs.* ≥50; 3) (pulmonary ARDS (pneumonia, aspiration, pulmonary contusion, near drowning) *vs.* extrapulmonary ARDS (non-pulmonary sepsis, trauma without pulmonary contusion, major surgery, multiple transfusions, traumatic brain injury, drug overdose, shock, other cause) (4) time of ARDS ≤36 h *vs.* >36 to <72 h; (5) mechanical ventilation ≤2 days, 3 to 4 days, ≥5 days. Effects on subgroups will be evaluated using the chi-square test for homogeneity. Statistical significance is defined as *P* < 0.05. All analyses will be carried out using the statistical software R (R Development Core Team, URL http://www.R-project.org - version 2.13) or STATA SE 11 for Windows (College Station, TX, USA).

### Ethical aspects

Each investigator center will submit the study protocol to its institutional Research Ethics Board (REB). The study should start only after being approved by the REB. Written informed consent will be obtained from a legal representative of all participants. This study is in compliance with Brazilian and international declarations.

### Trial organization and management

#### *Trial management team (TMT)*

A team based on the Research Institute HCor, São Paulo, Brazil will manage the trial on a day-to-day basis. The TMT is comprised by the chief investigator, a project manager, a statistician, and a computer programmer.

The responsibilities of the TMT include:

Planning and conducting the study: designing the protocol; designing the electronic case report forms (e-CRF); designing the operation guide; managing and controlling data quality; designing, testing, and maintaining the electronic data capture system; continuous data quality control; assisting the steering committee;

Managing the research centers: selecting and training the research centers; helping the centers prepare a regulatory report to be submitted to the REBs and assisting the centers with the submission; monitoring recruitment rates and the actions to increase recruitment; monitoring follow-up and implementing actions to prevent follow-up losses; auditing; sending study materials to the research centers; producing a monthly study newsletter; developing supporting material for the study;

Statistical analysis and research reporting: complete statistical analysis; helping to write the final manuscript.

#### *Trial steering committee (TSC)*

The TSC is responsible for the overall study supervision, assisting in developing the study protocol and preparing the final manuscript. All other study committees report to the TSC. The TSC members are investigators trained in designing and conducting randomized clinical trials, intensivists, respiratory therapists, and pulmonologists experienced in conducting multicenter randomized studies on ARDS.

#### *Trial centers*

Initially 80 centers would be invited to participate in the study, but the current goal is to involve 120 centers in the study. Details of the centers which accepted to participate in the trial at the time of this manuscript submission are given in the Appendix.

#### *Institutional support from the Brazilian Association of Intensive Care Medicine (Associação de Medicina Intensiva Brasileira (AMIB))*

The AMIB supports the ART study by means of the AMIB-Net. The AMIB-Net will assist with the selection and invitation of centers to participate in the ART, as well as facilitate the organization of meetings of researchers during national scientific meetings organized by the AMIB.

#### *Publication policy*

The ART study success depends on all its collaborators. Therefore, the primary results of the trial will be published under the name of ART Investigators. The contributions of all collaborators, their names and respective institutions, will be acknowledged in the manuscript. To safeguard the scientific integrity of the study, data from this study will be submitted to publication only after the final approval from the TSC.

#### *Data monitoring committee (DMC)*

The DMC is set up with independent epidemiologists and intensivists. The DMC is in charge of providing recommendations for the TSC of continuing the study as planned or discontinuing the recruitment based on evidence that the intervention causes increased mortality in the experimental group as compared to the control group. Interim analyses will be conducted after recruitment of approximately 33% and 66% of the sample. Based on these interim analyses, and, occasionally, on external evidence, the DMC shall decide whether there is evidence beyond a reasonable doubt that the treatment is clearly contraindicated in all patients or any subgroup. The criterion of evidence beyond a reasonable doubt is increased mortality at 28 days with the maximum lung recruitment strategy compared with the low PEEP strategy, with *P* <0.01. Otherwise, the TSC and other investigators will not be informed of the results of interim analyses. Considering previous evidence showing that: (1) early discontinuation of randomized trials due to benefits tends to produce biased estimates of effect (overestimation of the true effect), leading to erroneous medical guidelines and decisions [19]; (2) according to the ethical principle of non-maleficence, a new treatment should not be used until there is clear, objective evidence that it is beneficial; (3) clinical practice usually does not change unless there is fairly convincing evidence of the advantages of the new treatment, which would be undermined if the study is discontinued early due to benefits; the decision of early discontinuation of the experimental treatment due to benefits may not be advantageous for future patients, or may contribute to mislead guidelines [20]. For these reasons, early discontinuation of the study due to benefits of the experimental treatment is not planned.

## Discussion

ARDS is a common problem in intensive care associated with a very large in-hospital mortality rate, in spite of advances in therapy [3]. Maximum alveolar recruitment followed by PEEP titration is a relatively simple, inexpensive, and widely available intervention with potential to improve the prognosis of patients with ARDS. In most patients with ARDS, this strategy is able to keep open more than 90% of lung mass, improving oxygenation and preventing atelectrauma [13]. However, the effects of this strategy on patient important outcomes remain to be established. Therefore, evidence from well designed and conducted trials to solve this question is essential.

The ART was planned to be the largest randomized trial involving ARDS patients conducted to date. It will provide a precise and reliable estimate of the effect of alveolar recruitment and PEEP titration on survival of ARDS patients compared to the ARDSNet strategy, currently the evidence-based best approach for mechanical ventilation.

If our study finds that the maximum alveolar recruitment plus PEEP titration is not beneficial, this will play a role in changing medical practice since maximum alveolar recruitment associated with high levels of PEEP is routinely used by many intensivists. On the other hand, if the study demonstrates that maximum alveolar recruitment associated with PEEP titration increases survival in patients with ARDS, this will represent a valuable improvement for the treatment of ARDS patients.

## Trial status

The ART is currently ongoing in 103 sites in Brazil, Colombia, Italy, and Mexico. Enrollment started in December 2011 in one site. Now, 40 sites are actively screening for patients, and the remaining are undergoing REB evaluation. As of June 1, 2012, we had already enrolled 63 patients. We are inviting centers in other countries to join us.

## Appendix

The ART Investigators consists of:

Writing and Steering Committee: Alexandre B. Cavalcanti (Co-Chair), Otávio Berwanger, Érica A Suzumura, Marcelo BP Amato, Fernando S Tallo, Ederlon AC Rezende, José MM Telles, Edson Romano, Hélio P Guimarães, Marisa M Regenga, Luzia N Takahashi, Cassiano Teixeira, Roselaine P Oliveira, Vitor O Carvalho, Fredi A Díaz-Quijano, Carlos RR Carvalho (Co-Chair and Senior Investigator).Trial Management Team: Ale*x*andre B Cavalcanti, Érica A Suzumura, Otávio Berwanger, Alessandra A Kodama, Gisele FM Ribeiro, Matheus O Abreu, Ivonaldo M Oliveira.Data Monitoring Committee: Gordon Guyatt (Chair), Niall Ferguson, Stephen Walter.*Trial centers: *Brazil: Hospital de Urgências e Emergências de Rio Branco–HUERB, Rio Branco-AC: Márcia O. M. Vasconcelos, Valério J. Segundo, Íris L. Ferraz, Rosicley S. Silva; Hospital e Pronto-Socorro 28 de Agosto, Manaus-AM: Wilson de Oliveira Filho, Nelson B. Silva, Débora C. B. Heirel, Rodrigo R. Takatani, Jefferson A. Sousa Neto, Jerônimo C. B. Neto, Samara D. Almeida, Gauco Chamy; UNIMED Manaus, Manaus-AM: Wilson de Oliveira Filho, Graciliano J. L. Gonçalves Neto, Samara D. Almeida, Alysson P. Dias, Rozangela R. Silva; Fundação Hospital Adriano Jorge, Manaus-AM: Roberta C. Tavares, Márcia L. V. D. Souza, Janaína C. Decio; Hospital Santa Izabel - Santa Casa de Misericórdia da Bahia, Salvador-BA: Cyntia M. L. S. Lima, Fleury Ferreira Neto; Hospital Regional de Juazeiro - Gestão IMIP, Juazeiro-BA: Kátia R. Oliveira, Polyana P. L. C. Dias, André L. S. B. Brandão, Joroastro E. Ramos Jr, Paula T. Vasconcelos; Hospital Universitário Prof. Edgar Santos, Salvador-BA: Dimitri G. Flôres, Gilvan R. Pinheiro Filho, Isaac G. Andrade; Hospital Espanhol, Salvador-BA: Amadeu Martinez, Gustavo G. P. França, Lívia L. Monteiro, Emmanuel I. S. Correia, Wagner Ribeiro, Antonio J. Pereira, Wandalvo Andrade, Petrônio A. Leite, Gilvan R. Pinheiro Filho; Hospital Geral Roberto Santos, Salvador-BA: Dimitri G. Flôres; Hospital de Messejana, Fortaleza-CE: José E. Filgueira Feto, Marcelo A. Holanda; Hospital Regional de Samambaia, Brasilia-DF: Fábio F. Amorim, Silviano B. Margalho; Hospital Regional da Asa Norte - HRAN, Brasilia-DF: Sergio M. Domingues Jr, Claiton S. Ferreira, Cassia M. Ferreira, Livia A. Rabelo, Juliana N. Duarte, Fernando B. Lima, Inês A. L. Kawaguchi; Hospital Santa Luzia, Brasilia-DF: José A. Araújo Neto, Marcelo O. Maia; Hospital Santa Lucia, Brasilia-DF: Fabiano G. Correa; Hospital Anchieta, Brasilia-DF: Rubens A. B. Ribeiro; Centro Integrado de Atenção à Saúde – CIAS, Vitória-ES: Eliana Caser, Cora L. C. B. Moreira, Antonielen Marcilino, Jansen G. Falcão, Karinne R. Jesus, Leo Tcherniakovisk, Victor G. Dutra; Hospital Evangélico de Cachoeiro de Itapemirin, Cachoeiro de Itapemirim-ES: Marlus M. Thompson; Vitória Apart Hospital, Vitória-ES: Claudio Piras, Jonas Giuberti Jr, Albano S. Silva; Vila Velha Hospital, Vila Velha-ES: José R. P. Santos, Jorge L. Potratz, Ludmila N. Paula, Giovana G. Bozi, Bruno C. Gomes; Hospital das Clínicas - UFES, Vitória-ES: Paula F. Vassallo, Edson P. Rocha, Maria H. B. S. Lima; Hospital das Clínicas (UFG), Goiania-GO: Denise M. Ferreira, Fernanda A. F. Gonçalves, Sheila A. Pereira, Marciano S. Nobrega, Carlos R. Caixeta; Hospital Geral Tarquínio Lopes Filho, São Luiz-MA: Ana P. P. Moraes; UDI Hospital, São Luiz-MA: Alexandre G. R. Carvalho; Santa Casa da Misericórdia de Ouro Preto, Ouro Preto-MG: Janine D. Alves; Hospital Eduardo de Menezes, Belo Horizonte-MG: Frederico B. Carvalho, Fabiana B. R. Moreira, Claudia M. Starling, Wivian A. D. Couto; Fundação Hospitalar São Sebastião, Tres Corações-MG: Wesley S. Bitencourt; Hospital Cônego Monte Raso, Baependi-MG: Wesley S. Bitencourt; Hospital Municipal Odilon Behrens, Belo Horizonte-MG: Frederico B. Carvalho, Daniela C. Peixoto, Ivana L. V. Carvalho, Silvângela G. A. Silva, Livia R. S. M. Felizardo, Francine J. Magalhães Nascimento, Priscila J. C. D. Santos, Camila C. Zanta, Marcele F. Martins; Hospital São Lucas de Governador Valadares, Governador Valadares-MG: Sérgio A. Naves, Fabiano D. Silva, Gilberto Laube Jr; Santa Casa de Caridade de Diamantina, Diamantina, MG: Endi L. Galvão, Marcelo F. Sousa, Marcia M. F. Souza, Fernanda L. G. Carvalho; Hospital Santa Lúcia - Hospital do Coração de Poços de Caldas, Poços de Caldas-MG: Ricardo R. Bergo; Hospital Regional de Mato Grosso do Sul Rosa Pedrossian, Campo Grande-MS: Claudnei M. Rezende, Edys Y. Tamazato, Saturnino Campo Sarat Jr, Patrícia S. Almeida, Anthony G. Gorski; Hospital Universitário - Universidade Federal da Grande Dourados, Dourados-MS: Mirna Matsui, Ervin Eberhart Neto, Silmara H. Nomoto, Zildamara B. Lima, Alexandre S. Inagaki, Fernando S. U. Gil, Mario F. A. Araújo, Aline E. Oliveira, Tiago A. Correa, Angela Mendonça; Hospital de Clínicas Gaspar Vianna, Belém-PA: Helder Reis, Saul R. Carneiro; Carlos Castanelo, Edward Coelho Jr, Karine A. E. H. Amaral; Hospital Saúde da Mulher, Belém-PA: Leila R. M. Rego, Adenard F. C. Cunha, Williams F. Barra, Maurício Carneiro, Roseane A. Batista, Karina K. Zoghbi; Fundação Santa Casa de Misericórdia do Pará, Belém, PA: Nelma J. N. Machado, Reinaldo Ferreira, Pablo Apoena, Rosangela M. Leão; Hospital de Emergência Trauma Senador Humberto Lucena, João Pessoa-PB: Eliauria R. Martins, Marcelo E. Oliveira, Isaura Odir, Wladimy Kleber, Daniele Tavares; Hospital UNIMED João Pessoa, João Pessoa-PB: Eliauria R. Martins, Marcelo E. U. Araújo, Yuzeth Nóbrega Brilhante, Daniele C. C. Tavares, Wladmy Kleber, Waneska L. N. Carvalho, Geórgia F. P. Winveler; Hospital Alfa, Recife-PE: Aldir Chagas Filho, Raphael Ali Cavalcanti; Hospital Evangélico de Londrina, Londrina-PR: Cintia M. C. Grion, Andrezza T. J. B. Reis, Josiane Festti, Francielli M. P. Gimenez; Hospital Universitário Regional do Norte do Paraná, Londrina-PR: Cintia M. C. Grion, Alexandre S. Larangeira, Lucienne T. Q. Cardoso, Ana L. Mezzaroba, Thiago S. Giancursi, Ivanil A. M. Kauss; Hospital São Lucas/FAG, Cascavel-PR: Péricles A. D. Duarte, Tatiane C. Tozo, Priscila Peliser; Hospital Universitário Regional de Maringá, Maringá-PR: Almir Germano, Sanderland J. T. Gurgel, Sandra R. B. Silva, Cristina M Kuroda, Andrea Herek, Sergio S. Yamada; Hospital Santa Casa - Campo Mourão, Campo Mourão-PR: Paulo M. Schiavetto; Hospital Santa Tereza de Guarapuava, Guarapuava-PR: Natacha Wysocki, Rosely R. Matsubara; Hospital de Clínicas de Padre Miguel, Rio de Janeiro-RJ: João A. L. Sales Jr, Maria P. Laprovita; Hospital Prontocardio, Campos dos Goytacazes-RJ: Felipe M. Pena, Alexandre Sá; Clínica São Vicente, Rio de Janeiro-RJ: Arthur Vianna; Hospital Barra D’Or, Rio de Janeiro-RJ: Juan C. Verdeal, Glória A. Martins, Diamantino R. Salgado; Hospital Monte Sinai, Ariquemes-RO: Adalberto M. Coelho, Milena P. P. M. Coelho, Aline S. Morong, Rodolfo M. B. Poquiriqui, Ana P. Ferreira, Debora N. L. Lucena, Nathalia F. Marino, Monique A. Moreira, Cristiana C. S. Uratani; Hospital Estadual e Pronto Socorro João Paulo II, Porto Velho – RO: Marta A. Severino, Patrícia N. Silva, Luciana G. Medeiros, Francisco G. Chaves Filho, Daniela M. Q. S. Guimarães; Hospital Geral de Roraima, Boa Vista-RR: Valéria M. C. Rezende, Roberto C. C. Carbonell, Renata S. Trindade; Hospital Nossa Senhora da Conceição, Porto Alegre-RS: José A. S. Pellegrini, Márcio M. Boniatti, Moreno C. Santos, Rodrigo Boldo, Vanessa M. Oliveira, Viviane M. Corrêa, Wagner Nedel; Hospital Moinhos de Vento, Porto Alegre-RS: Cassiano Teixeira, Roselaine P. Oliveira, Felipe Schaich, Luciana Tagliari, Augusto Savi, Luis F. Schulz, Juçara G. Maccari; UTI Central - Irmandade Santa Casa de Misericórdia de Porto Alegre, Porto Alegre-RS: Roselaine P. Oliveira, Cassiano Teixeira, Gabriela M. Seeger, Rafael B. Foernges, Marcelo M. Rieder, Daniel A. Becker, Fabiano P. Broilo; UTI do Pavilhão Pereira Filho - Santa Casa de Misericórdia de Porto Alegre, Porto Alegre-RS: Patrícia Schwarz, André Alencastro, Paula Berto, Fabiane Backes, José A. S. Pellegrini; Hospital São Lucas da PUCRS, Porto Alegre-RS: Fernando S. Dias, Clarissa Blattner, Edna T. J. Martins, Nóris C. Scaglia; Hospital de Clínicas de Porto Alegre (HCPA), Porto Alegre-RS: Silvia R. R. Vieira, Karen F. Prado, Lea Fialkow, Cristiano Franke, Debora F. V. B. Vieira, Rafael B Moraes, Patrícia Schwarz, Leonardo S. Marques, João L. S. Hopf, Iuri C. Wawrzeniak, Tatiana H. Rech, Régis B. Albuquerque; Hospital Universitário São Francisco de Paula, Pelotas-RS: Márcio O. Guerreiro, Luciano O. Teixeira, Pedro L. Macedo, Marina P. Bainy, Edgard V. Ferreira; Hospital do Coração - Balneário de Camboriu-SC: Marcio A. Martins, Luciana A. S. Andrade; Hospital Universitário - UFSC, Florianópolis-SC: Fernando O. Machado; Hospital Nereu Ramos, Florianópolis-SC: Ana C. Burigo, Mariangela Pincelli, Lara Kretzer, Israel S. Maia; Hospital UNIMED Joinville, Joinville-SC: Rodrigo B. Cordeiro, Glauco Westphal, Milton Caldeira, Amanda S. Cramer, Michelli M. Dadam, Pierry O. Barbosa; Hospital São José, Joinville-SC: Milton Caldeira, Glauco Westphal, Caroline O. Brilenger, Marina B. W. Horner, Glauce L. Oliveira, Bruno C. Germiniani, Cristina Teixeira, Robson Duarte; Hospital Regional Hans Dieter Schmidt, Joinville-SC: Maria G. P. L. Assef, Deorgelis Rosso, Rodrigo Bigolin, Raquel Vanzuita; Associação Hospitalar e Maternidade Cônsul Carlos Renaux, Brusque-SC: Márcio A. Martins; Hospital Primavera, Aracaju-SE: Luiz F. A. Prado, André L. V. Oliveira, Diego L. Reis, Mirene O. Morais, Rafael S. Bastos, Hericalizandra S. R. Santana, Alline O. Silva, Lucas A. P. Cacau, Marília S. Almeida; Hospital de Urgência de Sergipe - HUSE, Aracaju-SE: Hugo Schlebinger Canavessi, Eduardo E. F. Nogueira, Caio L. P. Pavia, Diego L. Reis, José F. Araujo, José A. Lira, Esteban C. Nienstedt, Thiago C. Smith; Hospital do Coração - HCor, São Paulo-SP: Edson Romano, Marcelo Romano; Marisa M. Regenga, Dalton Barros, André F. Costa, Luzia Takahashi, Vinicius Werneck, Jorge Farran, Lilian A. Henriques, Claudia Miura; UTI da Clínica Médica - Hospital São Paulo – UNIFESP, São Paulo-SP: Renato D. Lopes, Letícia S. Vendrame, Hélio P. Guimarães, Priscila Sandri, Marcela S. Galassi; UTI Respiratória - Hospital das Clínicas da FMUSP, São Paulo-SP: Carlos R. R. Carvalho, Marcelo B. P. Amato, Carlos Toufen Jr, Roberta R. S. Santiago, Adriana S. Hirota; UTI Clínica - Hospital das Clínicas da FMUSP, São Paulo-SP: Marcelo Park, Luciano C. P. Azevedo; UAC I e UAC II - Hospital das Clínicas da FMUSP, São Paulo-SP: Luiz M. Malbouison; UTI da Nefrologia - Hospital das Clínicas da FMUSP, São Paulo-SP: Maristela C. Costa; UTI Clínica do Pronto Socorro - Hospital das Clínicas da FMUSP, São Paulo-SP: Leandro Taniguchi; UTI Cirúrgica - Hospital das Clínicas da FMUSP, São Paulo-SP: Carlos E. Pompílio; Hospital Moyses Deutsch (M’Boi Mirim), São Paulo-SP: Claudio Baruzzi, Ana H. V. Andrade, Elisabete E. Taira, Bruno Taino, Clezio S. Oliveira, Anselmo C. Silva; Hospital do Servidor Público Estadual, São Paulo-SP: Alexandre Ísola, Ederlon Rezende, Ricardo G. Rodrigues, Vivian P. L. Rangel, Sergio Luzzi, Ivens W. S.Giacomassi; Hospital e Maternidade São Camilo - Unidade Pompeia, São Paulo-SP: Antonio P. Nassar Jr, Ana R. Souza; Hospital São Luiz - Unidade Itaim, São Paulo-SP: Luciana Rahal, Andre L. Nunes, Fabio Giannini, Brena Menescal, Jussara E. P. Morais, Diogo Toledo; Hospital São Luiz - Unidade Anália Franco, São Paulo-SP: Rafaela D. Morsch, Thalita Merluzzi, Denise S. Amorim, Ana C. A. G. Bastos, Patrícia L. Santos, Sabrina F. Silva, Raquel C. N. Gallego, Gheisa D. Santos; AC Camargo, São Paulo-SP: Mauro Tucci, Ramon T. Costa, Lucio S. Santos, Sergio E. Demarzo; Hospital Sírio Libanês, São Paulo-SP: Guilherme P. P. Schettino, Luciano C. P. Azevedo, Vivian C. Suzuki, Ana C. L. Patrocinio, Mariana L. Martins, Denise B. V. G. Passos; Hospital da Luz, São Paulo-SP: Sylas B. Cappi; Hospital SãoLuiz - Unidade Morumbi, São Paulo-SP: Iran Gonçalves Jr; HCRP-FMRP-USP, Ribeirão Preto-SP: Marcos C. Borges, Wilson Lovato, Marcel V. Tavares, Daniela Morales, Luis A. M. W. Machado, Franciele C. C. Torres, Tania M. Gomes, Rodrigo B. Cerantola; UTI do Pronto Socorro - Hospital São Paulo - UNIFESP, São Paulo-SP: Aécio Góis, Thiago Marraccini; Kathia Margarida, Eulália Cavalcante; UTI Anestesiologia - Hospital São Paulo -UNIFESP, São Paulo-SP: Flávia R. Machado, Bruno F. Mazza, Heloisa B. Rossetti Santana, Vanessa M. F. Mendez, Patricia A. Xavier, Melina V. Rabelo, Fabiana R. Schievano, Walkyria A. M. Pinto, Renata S. Francisco, Elaine M. Ferreira; Instituto de Oncologia Pediátrica GRAACC - UNIFESP, São Paulo-SP: Dafne C. B. Silva, Rodrigo G. Arduini; Hospital e Maternidade São Cristóvão, São Paulo-SP: José R. Aldrighi, Andreson F. Amaro; Hospital José Soares Hungria, São Paulo-SP: Katia A. P. Conde; Hospital Municipal Professor Doutor Alipio Correa Neto, São Paulo-SP: Cesar A. Pereira, Elcio Tarkieltaub, Wilson R. Oliver, Erika G. L. Guadalupe, Paulo S. C. Acerbi, Carlos I. Tomizuka, Tatiana A. Oliveira, Nadia N. Geha; Hospital Universitário São Francisco, Bragança Paulista-SP: Giovana C. Mecatti, Maysa Z. R. Piovesan, Maria C. Salomão; Hospital UNIMED Araçatuba-SP: Marcelo S. Moreno, Vinicius N. Orsatti, Whiniton Miranda; Hospital Bandeirantes, São Paulo-SP: Alexandre Ray, André Guerra, Mario L. A. Baptista Filho; Hospital Geral São Mateus Dr. Manoel Bifulco, São Paulo-SP: Firmino H. Ferreira Jr, Edésio Viera Filho, Regina A. Canzi, Adriana F. T. Giuberti, Melissa C. M. Garcez; Hospital Escola Padre Albino - Faculdades Integradas Padre Albino - Medicina, Catanduva-SP: Jorge L. Valiatti, Francisco C. Lucca, Júlio C. Fornazari; Santa Casa de Misericórdia de Tatuí, Tatuí-SP: Vivian M. Irineu, Wladmir F. Saporito, Lucas Frare; Hospital Alemão Oswaldo Cruz, São Paulo-SP: José P. Ladeira, Ricardo Cordioli, Marcelo A. Pedro, Andrea D. Sala, Edmundo O. Suguitani, Priscila Kazue, Luiz R. C. Oliveira, Rodrigo M. Infantini; Instituto de Infectologia - Emílio Ribas, São Paulo, SP: Fabrício R. T. Carvalho, Lucia C. Andrade, Hélio P. Guimarães; Hospital de Clínicas da UNICAMP, Campinas-SP: Thiago M. Santos, César V. Carmona, Luciana C. Figueiredo, Antonio Falcão, Desanka Dragosavak; Hospital das Clínicas Luzia de Pinho e Melo - SPDM, Mogi das Cruzes-SP: Wilson Nogueira Filho, Maria C. Lunardi, Roberto Lago, Ciro Gatti, Tatiana M. Chiasso, Grazielle O. Santos, Aline C. F. Silva, Arthur C. Araujo; Irmandade Santa Casa de Misericórdia de Mogi Guaçu, Mogi Guaçu - SP: Izadora B. Ornellas, Vitor M. Vieira; Instituto do Câncer do Estado de São Paulo - ICESP, São Paulo-SP: Ludhmila A. Hajjar, Adelaide C. Figueiredo; Hospital Maternidade UNIMED Leste Paulista, São João da Boa Vista-SP: Vitor M. Vieira, Bruna Damasceno. Colombia: Fundación Cardiovascular de Colombia, Bucaramanga: Camilo Pizarro; Los Comuneros Hospital Universitario de Bucaramanga, Bucaramanga: Alfredo Hinestrosa; Organización Latinoamericana para el Fomento de la Investigación en Salud - OLFIS, Bucaramanga: Fredi A. Diaz-Quijano. Mexico: Medica Sur, Ciudad de México-DF: Sandra M. C. García L., Octavio González C., Edgard Díaz S. Italy: Azienda Ospedaliera Universitaria P. Giaccone, Palermo: Santi M. Raineri, Andrea Cortegiani.

## Competing interests

The authors declare that they have no competing interests.

## Authors’ contributions

ABC, EAS, OB, MBPA, and CRRC conceived the study, participated in its design and coordination, and helped to draft the manuscript. FST, EACR, JMMT, ER, HPG, MMR, LNT, CT, RPO, VOC, and FADQ participated in the study design and helped to draft the manuscript. All authors read and approved the final manuscript.

## *Sources of funding*

This study is funded by the Hospital do Coração (HCor) as part of the Program ‘Hospitais de Excelência a Serviço do SUS (PROADI-SUS)’ in partnership with the Brazilian Ministry of Health.
